# Castasterone, a Plant Steroid Hormone, Affects Human Small-Cell Lung Cancer Cells and Reverses Multi-Drug Resistance

**DOI:** 10.3390/ph16020170

**Published:** 2023-01-23

**Authors:** David Sadava, Shiuan Chen

**Affiliations:** Department of Cancer Biology and Molecular Medicine, Beckman Research Institute, City of Hope, 1500 E. Duarte Rd., Duarte, CA 91010, USA

**Keywords:** small-cell, lung cancer, castasterone, plant hormone, multi-drug resistance

## Abstract

Small-cell lung cancer (SCLC) has a dismal prognosis, in part because of the development of multi-drug resistance. Castasterone (CAS) is the metabolic precursor of the plant steroid hormone epibrassinolide (EB). In some plants, EB accounts for the total hormone activity, whereas in other plants, CAS is the active form. The effects of CAS, a BR present in most plants, on animal cells in general and cancer cells in particular have not been described. Here, we report the effects of CAS on drug-sensitive (H69) and drug-resistant (VPA17) SCLC cells. CAS was equally cytotoxic to both cell lines (IC_50_ = 1 μM), indicating a lack of cross-resistance. Pre-incubation of VPA17 cells with CAS for 96 h reversed drug resistance to etoposide and doxorubicin. Synergism between CAS and EB, as well as with chemotherapy drugs, was investigated by exposure of VPA17 cells to 1:1 ratios of CAS and the other drugs at the respective IC_50_ values, with dilutions at 0.25 to 2.0 × IC_50_ and determination of the combination index (CI). CAS and EB were additive, indicating that the two drugs act on the same pathway, whereas CAS–etoposide (CI = 0.77) and CAS–doxorubicin were synergistic, indicating that CAS and the two chemotherapeutic drugs act on different pathways. Apoptosis in SCLC cells was measured by immuno-detection of single-strand DNA breaks. Following 96 h incubation of SCLC H69 cells in CAS, the level of DNA breaks was similar to measurements made after incubation in EB and etoposide, indicating that CAS is pro-apoptotic. Incubation of SCLC cells in CAS led to a time-dependent reduction (by 80%) in the transcriptional activator β-catenin. These data indicate that CAS may act via *Wnt* signaling. Taken together, our study reveals that CAS is pharmacologically active in both drug-sensitive and drug-resistant SCLC cells.

## 1. Introduction

Small-cell lung cancer (SCLC) is a neuroendocrine carcinoma that accounts for approximately 13% of all lung cancers. SCLC is very aggressive, with rapid spread to distant metastases and a poor prognosis. Most patients present at diagnosis with stage IV disease and a dismal prognosis [1]. Because of disseminated disease, treatment is by cytotoxic chemotherapy [2]. Despite the initial sensitivity of the tumor to chemotherapy, multi-drug resistance develops, and few patients survive beyond 10 years [3]. In contrast to non-small-cell lung cancer, research on SCLC is only beginning to yield results pointing to molecular-based therapies [4].

Brassinosteroids (BRs) are plant steroid hormones first identified in the pollen of rape (*Brassica napus)* with activity in promoting cell elongation [5]. Since then, BRs have been found throughout the plant kingdom [6], with many effects on plant growth, development, and stress response [7]. In contrast to animal hormones, which are typically made in a specific glandular location and exert effects elsewhere in the organism, plant hormones are usually made in many locations and can have local as well as distant effects. BRs are synthesized in all plant organs, in higher concentrations in the tissues of young plants. They are therefore consumed as part of the human diet, where their effects are unknown.

BRs are synthesized in a multi-step pathway, best described by mutation and biochemical analyses in the model angiosperm plant *Arabidopsis thaliana* [8]. The end-product is the most common BR, epibrassinolide (EB). Molecular genetic studies of *Arabidopsis* have revealed the molecular signaling mechanism of EB activity [9]. The similarity of this mechanism to *Wnt* signaling in animal cells, including the presence of a GSK-like kinase in both pathways, has led to studies of the effects of EB on cancer cells, where the *Wnt* pathway is active. Molecular evidence indicates that EB acts on SCLC cells via *Wnt* signaling [10].

**Figure 1:**
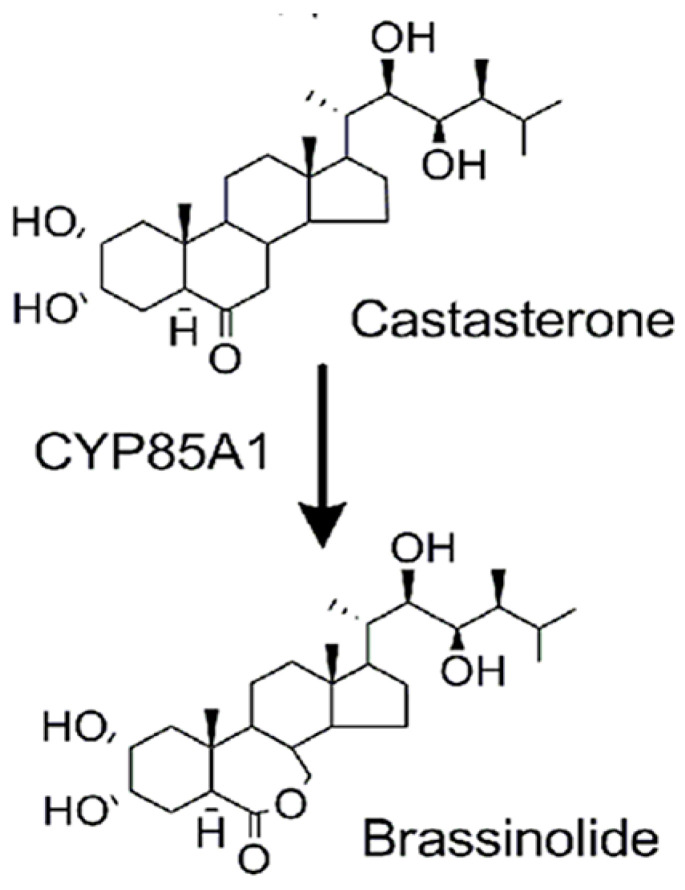
Role of castasterone in the synthesis of the plant hormone brassinolide.

The final step in EB biosynthesis is the conversion of castasterone (CAS) to EB [11]. This reaction is catalyzed by CAS-C-6 oxidase CYP85A1, also called BL synthase (Figure 1). While the gene encoding this enzyme occurs in most dicotyledonous plants (e.g., *Arabidopsis),* it is not present in most monocotyledons (e.g., rice) [12], nor is this enzyme present in animal cells, from the lack of the oxidase-encoding gene in animal genomes, including humans. In plants lacking the oxidase gene, the active BR is CAS. While CAS appears to act in both monocotyledons and dicotyledons via the kinase signaling pathways described above, the effects of CAS on animal cells are not known. Reported evidence that EB affects SCLC cells, as well as several other cancer cell types [13,14], prompted us to investigate the possible anti-cancer effects of CAS, another active BR in plants. We chose SCLC because of the need for new therapeutic approaches and the prominence of multi-drug resistance.

## 2. Results

### 2.1. Cytotoxicity of Castasterone

The cytotoxic effect of castasterone (CAS) was evaluated on drug sensitive- (H69) and multi-drug resistant (VPA17) SCLC cells. Cytotoxicities were similar (*p* < 0.05) at IC_50_ = 1.0 μM for both cell lines. Cytotoxicity was not evident for CAS on BEAS-2 normal lung epithelial cells, IC_50_ > 40 μM.

### 2.2. Reversal of Drug Resistance by CAS

Following pre-incubation of VPA17 drug-resistant SCLC cells in 0.5 μM CAS, resistance to etoposide and doxorubicin was significantly reversed (Table 1). Pre-incubation of drug-sensitive (not resistant) H69 cells with CAS did not affect the cytotoxicities of the two chemotherapeutic drugs.

### 2.3. Combinations of CAS and Chemotherapeutic Drugs

To determine possible interactions at the mechanistic level, drug-resistant VPA17 cells were separately exposed to combinations of CAS and the chemotherapeutic drugs, etoposide and doxorubicin, as well as EB, its metabolic product in plants, at a constant 1:1 ratio (1 × IC_50_: 1 × IC_50_) at dilutions from 0.25 to 2.0. Following calculation of cytotoxicities, the combination index was calculated [15]. For etoposide and doxorubicin, there was synergism with CAS, whereas with EB, the effect was additive (Table 2).

### 2.4. CAS Induction of Apoptosis: DNA Fragmentation

Apoptosis was measured in SCLC cells after CAS exposure by ELISA for single-strand DNA breaks. Compared to untreated cells, there was a significant increase in DNA fragmentation induced by CAS (Table 3). This increase was similar to those induced by the brassinosteroid EB, as well as the chemotherapeutic drug etoposide. The assay was validated by cell extracts treated with S1 nuclease to induce DNA fragmentation (OD_405_ = 1.74 ± −0.77) and by DNA containing single-strand breaks (OD_405_ = 1.85 ± 0.22).

### 2.5. CAS Effect on β-Catenin Concentration

**Figure 2:**
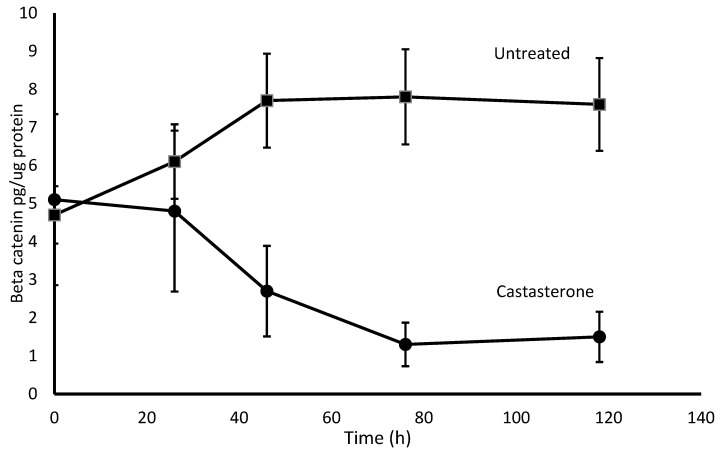
Time-dependent effect of castasterone on β-catenin in SCLC cells.

To investigate the intracellular pathway in cells affected by CAS, β-catenin was measured in H69 SCLC cell extracts. Compared to untreated cells, there was a time-dependent reduction of β-catenin in CAS-exposed cells (Figure 2). Similar results were obtained with VPA17 cells.

H69 cells were incubated without (untreated) or with 0.5 μM castasterone, and β-catenin and protein were measured at the indicated times in culture. Shown are the means (±S.D.) of the three experiments.

## 3. Discussion

Lung cancer is the leading cause of cancer deaths, and most patients die from a drug-resistant disease [16]. SCLC, while less than 15% of all lung cancers diagnosed, is particularly harmful, as it grows and spreads rapidly and has been insensitive to molecularly targeted therapies that have shown some benefit in other lung cancers [3]. BRs are widespread plant polyhydroxy steroid hormones, with over 70 different BRs identified [17], and are consumed in the human diet. Epibrassinolide (EB) is the most common and well-studied BR. While the effects of EB have been reported on animal cells (e.g., [10,13,14]), this is the first report on the effects of the other significantly active BR in plants, CAS, on animal cells, particularly cancer cells.

CAS is the final precursor to EB in the biosynthesis pathway in plants (Figure 1). In our experiments, CAS was cytotoxic to both drug-sensitive H69 and VPA17, multi-drug-resistant SCLC cells at equivalent IC_50_ concentrations (1 μM), indicating that the drug resistance mechanism does not affect the effect of CAS. These results are similar to the cytotoxicity of EB in these SCLC cells, where the reported IC_50_ was 2 μM. In addition, CAS cytotoxicity was not measurable in normal lung epithelial cells, indicating tumor cell specificity. Of additional importance for possible clinical use, pretreatment of VPA17 cells with CAS largely restored drug sensitivity (Table 1). In addition, our studies on drug combinations have shown synergism between CAS and to drugs commonly used in SCLC chemotherapy, etoposide and doxorubicin [1,2,3] (Table 2). This indicates that CAS acts on different biochemical pathways than the two drugs, which affect topoisomerase II and DNA, respectively. As an important control to these experiments, the effect of CAS and EB was additive, indicating that the two BRs act on the same pathway. It is not known if CAS exhibits synergy with drugs targeted to other cellular pathways.

Our initial experiments on the mechanism of CAS effects on SCLC cells indicate that it may act in a similar fashion to EB [10]. First, CAS was pro-apoptotic, as shown by the induction of single-stranded DNA breaks (Table 3). Second, CAS caused a time-dependent reduction in the concentration of β-catenin-treated SCLC cells (Figure 2). Increased β-catenin is a hallmark of the *Wnt* signaling pathway [18] and this pathway is activated in many cancers, including SCLC [19]. Our data indicate that CAS acts by negatively regulating *Wnt* signaling.

A recent review summarized the various mechanisms proposed for the effects of natural and synthetically modified BRs on human cells of various types [14]. These studies have included investigations of upstream (e.g., receptors) and downstream (altered gene expression and resulting cellular phenotype) effects. The evidence for binding of natural BRs to known steroid receptors in normal and cancer cells is either indirect or, in the case of the estrogen receptor, conflicting. Downstream effects on the activation of the genes involved in apoptosis and cell cycle stimulation have been reported for breast cancer and prostate cancer cell lines, with the signaling pathways unclear. Our data indicate that in SCLC cells, CAS may interact with *Wnt* signaling, a different pathway than previously reported.

In summary, our results indicate that CAS warrants further exploration in the treatment of drug-resistant SCLC.

## 4. Materials and Methods

### 4.1. Cells

NCI-H69 SCLC cells (ATCC, Manassas, VA, USA) were authenticated by ATCC-Promega and grown at 37 °C and 5% CO_2_ in suspension culture in AIM-V serum-free medium (Thermo-Fisher, Carlsbad, CA, USA). Multi-drug resistant VPA17 cells were derived from H69 cells by selection in etoposide [10]. The VPA17 cells were resistant to etoposide (10-fold) and doxorubicin (8-fold). The doubling time of both cell lines was 30 h. The medium was changed every 4 days. BEAS-2 normal lung epithelial cells were grown in DMEM-F12 supplemented with 10% fetal bovine serum.

### 4.2. Cytotoxicity and Reversal of Resistance

Castasterone (CAS) was obtained from Yuhao Chemical Technology, Hangzhou, China, and etoposide (ETOP), doxorubicin (DOX), and epibrassinolide (EB) were obtained from Sigma-Aldrich, St. Louis, MO, USA. These molecules were dissolved in DMSO and stored for up to 3 mo at −20 °C. Drugs were added to logarithmically growing cells in 0.2–10 mL AIM-V medium containing 10^4^ cells/mL. After 4–6 d incubation, cell counts were made by a hemacytometer, and live cell counts were validated by trypan blue exclusion. The experiments were carried out at least in triplicate. IC_50_ was defined as the concentration of added molecule that reduced treated cell cultures by 50% compared to cells incubated in 2% DMSO. In experiments involving pre-incubation, the cells were incubated in 0.5 × IC_50_ for 96 h, washed once with fresh AIM-V medium, and then tested for cytotoxicity after 96 h incubation.

### 4.3. Combination Studies with CAS, EB, and Chemotherapeutic Drugs

Synergism, additivity, and antagonism between the pairs of molecules were investigated using VPA17 cells [15]. Briefly, the cells were incubated in 1:1 ratio of the IC_50_ values of the molecule pairs in combination at 0.25, 0.5, 1.0, and 2.0 × IC_50_. After 120 h, cytotoxicities were determined and the combination index (CI) for the molecular pair was calculated using Calcusyn software (Biosoft, version 2.0).

### 4.4. Apoptosis Analysis by DNA Breaks

Apoptosis was determined by quantification of single-stranded DNA breaks (Novus Biologicals, Centennial, CO, USA). VPA17 cells (10^4^/mL) were incubated in 120 h in 0.5 × IC_50_ of CAS, EB, or ETOP and then fixed in methanol. Following drying of the cells to a microtiter plate, they were treated with formamide for 20 min followed by heating at 65 °C for 30 min to denature the DNA. Single-strand DNA breaks were determined by ELISA using a mouse monoclonal antibody to the breaks, followed by an HRP-coupled anti-mouse antibody. HRP was quantitated enzymatically using a peroxidase substrate.

### 4.5. β-Catenin Assay

β-catenin was determined quantitatively in cell extracts by ELISA (Enzo Life Sciences, Farmingdale, NY, USA). Following lysis of 5 × 10^5^ cells after treatment as indicated in in the Results section, double antibody ELISA was performed using a rabbit anti-human β-catenin antibody fixed to a microtiter plate followed by an HRP-coupled goat anti-rabbit antibody. HRP was detected spectrophotometrically. Human β-catenin was used as standard. The protein concentration was determined by the Bradford test (BioRad, Hercules, CA, USA), using bovine serum albumin as standard.

## Figures and Tables

**Table 1:** Reversal of multi-drug resistance by castasterone.

Condition	H69 (IC_50_, μM)	VPA16 (IC_50_, μM)
Etoposide, no preinc.	0.65 ± 0.35	6.70 ± 2.44 ^a^
Etoposide, CAS preinc.	0.57 ± 0.30	1.59 ± 0.98 ^b^
Doxorubicin, no preinc.	0.04 ± 0.02	0.32 ± 0.20 ^a^
Doxorubicin, CAS preinc.	0.04 ± 0.03	0.12 ± 0.03 ^b^

The cells were preincubated as noted in 0.5 μM CAS for 96 h, and then tested for cytotoxicity for 120 h in the drug as indicated. ^a^ Significantly different (*p* < 0.05) by *t*-test comparing H69 and VPA17 cells under the stated condition. ^b^ Significantly different (*p* < 0.05) by *t*-test comparing VPA17 cells with or without CAS pre-incubation.

**Table 2:** Combination index (CI) for castasterone (CAS), epibrassinolide (EB), and chemotherapy drugs.

Condition	ED75
CAS and Etoposide	0.77
CAS and Doxorubicin	0.85
CAS and Epibrassinolide	1.07

The SCLC VPA17 cells were exposed to 1:1 ratios of CAS and each of the other molecules at the IC_50_ values, with dilutions of 0.5–2.0 × IC_50_. After 120 h incubation, cytotoxicity was measured, and the combination index was calculated.

**Table 3:** DNA fragmentation induced by castasterone (CAS).

Condition	Single-Strand DNA Breaks, OD_405_
Untreated	0.54 ± 0.19
Castasterone	1.29 ± 0.47 ^a^
Epibrassinolide	1.38 ± 0.21 ^a^
Etoposide	1.35 ± 0.30 ^a^

The VPA17 SCLC cells were incubated for 96 h as noted in CAS (0.5 μM), EB (1.0 μM), or ETOP (3 μM). DNA breaks were measured by ELISA and expressed as OD_405_ nm ± S.D., means of four experiments. ^a^ Significantly different by *t*-test (*p* < 0.05) comparing the noted condition with the untreated controls.

## Data Availability

Data are contained within the article.
